# Pass or fail: Teachers’ experience of assessment of postgraduate critical care nursing students’ competence in placement. A qualitative study

**DOI:** 10.1186/s12912-024-01951-8

**Published:** 2024-05-23

**Authors:** Line J. Øvrebø, Dagrunn Nåden Dyrstad, Britt Sætre Hansen

**Affiliations:** 1 Faculty of Health Sciences, Department of Caring and Ethics, University of Stavanger, Postbox 8600, Stavanger, 4036 Norway; 2 Faculty of Health Sciences, Department of Quality and Health Technology, University of Stavanger, Stavanger, Norway

**Keywords:** Critical care nursing, Postgraduate education, Placement, Competence, Assessment, Assessment documents

## Abstract

**Background:**

Learning in placement is essential to postgraduate critical care nursing students’ education. Assessment of students’ competence in placement is important to ensure highly qualified postgraduate critical care nurses. The placement model applied in Norway involves students being assessed by a preceptor in practice and a teacher from the university. The teacher has a more distant role in placement, and the aim of this study was to explore how the teachers experience the assessment of postgraduate critical care nursing students’ competence in placement. Additionally, to explore the content of assessment documents used for postgraduate critical care nursing placement education in Norway.

**Methods:**

This study has a qualitative design with main data collection from individual interviews with 10 teachers from eight universities and colleges in Norway. Additionally, we performed a document analysis of assessment documents from all 10 universities and colleges providing postgraduate critical care nursing education in Norway. We followed the Consolidated Criteria for Reporting Qualitative Research.

**Results:**

The teachers experienced the assessment of postgraduate critical care nursing students’ competence in placement as important but complex, and some found it difficult to determine what critical care nursing competence is at advanced level. A thematic analysis resulted in one main theme: “Teacher facilitates the bridging between education and practice.” Furthermore, three themes were identified: “Assessment based on trust and shared responsibility”; “The teacher’s dual role as judge and supervisor”; and “A need for common, clear and relevant assessment criteria”.

**Conclusions:**

Teachers have a key role in placement as they contribute to the bridging between education and practice by providing valuable pedagogical and academic input to the assessment process. We suggest that more teachers should be employed in joint university and clinical positions to enhance the collaboration between practice and education. Clear and relevant assessment criteria are essential for providing assessment support for both students and educators. Education and practice should collaborate on developing assessment criteria. Further, there is a need to collaborate on developing, both nationally and internationally, common, clear, relevant and user-friendly assessment tools.

**Supplementary Information:**

The online version contains supplementary material available at 10.1186/s12912-024-01951-8.

## Background

Nurses’ competence is crucial to provide high-quality care to patients, and nurses with advanced and specialized expertise, like postgraduate critical care nurses (CCNs), can affect the treatment capacity of the health service, reduce intensive care unit length of stay, time to treatment and ameliorate costs [1–5]. CCNs need to integrate advanced theoretical knowledge with interpersonal and practical skills to take care of critically ill patients [6, 7]. Learning in placement is essential to nursing education, however research confirms that there are concerns regarding the educational quality of placements and nursing students’ fitness for practice upon graduation [8, 9]. To ensure a certain level of knowledge and skills, assessment of student competence is an important part of the education in placement [10–14]. Nurse educators play a key role in the assessment approaches [15], but the complexity of assessment can be challenging for educators [13, 16, 17]. Moreover, as postgraduate students are already qualified nurses, it can be difficult to assess which nursing competency is at a more advanced level [18]. According to Mårtensson et al. [19] postgraduate education programmes in health professions need to assess their students in relation to the expected standards and criteria upon programme completion, and the importance of having valid and reliable assessment is clear.

A common definition of competence is what individuals know or can do in terms of knowledge, skills, and attitude. A more holistic approach also involves the student’s ability to use theory, judgment, critical thinking, and professionalism [19]. Ääri et al. [20] presented competence in critical care nursing (CCN) in two domains; clinical competence and professional competence where the clinical competence included the knowledge- and skill base. The professional competence in CCN is perceived as difficult to describe, but terms like: teamwork, decision-making, being able to manage situations and care for the patients beyond the technical aspects, in addition to personal maturity and having a good attitude, are used to describe core competencies [2, 5, 7, 11, 20]. In this article the term competence will be used as a collective term for clinical and professional competence.

The purpose of assessment is to provide feedback to the students on their ability to perform the required skills and competencies. Assessment can be formative to monitor and give feedback on progress, or summative to indicate a final level of achievement [10, 21]. Assessment methods and requirements probably have a strong influence on what students learn, thus there should be a constructive alignment between assessment criteria and functional ability [22, 23]. As the concept of assessment contains different perspectives, the assessment of student`s competence in placement should contain both the student`s self-assessment and preceptor and teacher`s assessment [19, 24, 25]. The student`s self-assessment can be a consciousness-raising exercise for the students to become aware of their competence level and behavior. Assessment can also be done by grading practical, oral or written coursework against a set of specified criteria or in relation to the achievements of a group of people. Assessment that involves discussing performance with the student, may better reveal the student’s competence level [21].

Postgraduate nursing education can be organized either as a hospital-based specialization program, residency programs offered by healthcare institutions or as a university-based program [26–29]. Both theoretical and practical preparation are key components of nursing education [13]. However, CCN education programs vary worldwide from weeks to years with a master’s degree [2, 30], and it is difficult to establish the exact time and level when nurses are considered qualified CCNs [31]. Students of postgraduate courses are registered nurses with various professional background and work experiences [27]. Some students may have work experience from intensive care units prior to starting CCN education, whereas other students have no experience working with critically ill patients. Universities and colleges are responsible for graduating CCN students with high theoretical and practical competencies, nevertheless a review study by Øvrebø et al. [24] found great variation as to how the postgraduate CCN students’ competence in placement are assessed and variation in assessment requirements.

This study was conducted in Norway where CCN education is a postgraduate university or college program of optional 90 or 120 credits (master’s degree). Attendance requires a bachelor’s degree in nursing and at least two years ‘clinical experience. The program consists of at least 28 weeks supervised placement in intensive care unit (ICU) and theoretical education following a national curriculum. Assessment of CCN students’ competence during placement includes a partnership where CCN students, teachers, and preceptors collaborate on confirming the students’ achieved competence. The CCN students are supervised and assessed by a preceptor who is working as a CCN in practice and followed up by a teacher from the university or college. The teacher is responsible for the assessment process and provides information and support to preceptors and CCN students without engaging directly in patient care. During a placement period there should be at least three formal meetings between the CCN student, preceptor, and teacher. The first meeting is to clarify expectations, at mid-term the meeting focuses on formative assessment, and the last meeting is a summative assessment of the students’ competence. Although the teacher does not observe student skills in practice, the teacher will meet the students in reflection groups, simulation- and skills training in addition to the three planned meetings. Prior to the assessment meetings the students send a written self-assessment to the preceptor and teacher.

Helminen et al. [32] described the teacher’s role in Finland as similar to the Norwegian context. However, according to Immonen et al. [13] the role of nurse educators in the assessment of nursing students’ competence in placement varies internationally. In some countries, teachers from universities and colleges take the role of clinical facilitators and actively guide students during the placement period, but in several European countries the role of teachers in placement has decreased. Löfmark et al. [33] states that the role of nursing teachers in placement has changed from the traditional role of clinically skilled practitioner to a more distant, multifaceted and unclear role in the placement context. The transfer of education from hospitals to universities and colleges has led to an academization of teachers who are to a greater extent distant from practice [33–35]. Hence, there is a need to investigate the teachers’ role in relation to assessment of students in placement.

Challenges concerning the assessment of nursing students during placements have been reported previously [10, 12, 13, 24, 36], however to our knowledge few studies have investigated the assessment of postgraduate CCN students from the perspectives of the nursing teachers and content of assessment documents. Assessment of postgraduate students’ competence is different from that of pre-registration student nurses, and there is a need for research on how the teachers experience their role in the assessment of postgraduate student`s competence in placement. Further it is necessary to examine the content of assessment documents as there are no national or international common criteria, and the assessment documents are essential for the assessment process.

For the rest of this article postgraduate CCN students are referred to as CCN students, CCN supervisors in placement as preceptors, and nursing teachers as teachers.

## Methods

### Aim

The aim of this study was to explore how teachers experience the assessment of postgraduate CCN students’ competence in placement. Furthermore, we wanted to explore the content of assessment documents used for placement courses. The following research questions were developed:

- What is the teacher’s assessment of CCN students’ competence in placement based on?
- How do teachers experience their role in the assessment of CCN students’ competence in placement?
- How do teachers experience the use of assessment documents, and what is the content of assessment documents for placement courses?

### Design

We used a qualitative research design with individual interviews and document analysis. This design was suitable because we wanted to gain a deeper understanding of the teachers experiences on the assessment of students’ competence and content of the assessment documents [37]. The consolidated criteria for reporting qualitative research (COREQ) checklist were used to report the findings [38].

### Setting and sample

In Norway, CCN teachers must be registered CCNs with a completed master’s or PhD-level degree. Additionally, it is required that they have completed pedagogical studies within two years of employment. Teachers are mainly employed by the universities and colleges. However, a few teachers are employed in joint positions between hospitals and universities/ colleges.

We conducted a purposive sampling of teachers to participate in the interviews with the aim to obtain variation regarding workplace and experience [37]. The inclusion criteria were teachers with experience of assessing CCN students in placement, no specific exclusion criteria were set. To recruit participants, we contacted the managers of all the 10 postgraduate/master’s CCN programs in Norway. The managers were asked to communicate the request to participate in the study to possible participants. We received contact information from the managers to 18 teachers working at eight different universities and colleges. All the 18 teachers were invited to participate, and 13 agreed to be interviewed. However, three of the teachers withdrew before the interviews took place, and explained it was due to workload. This left us with 10 teachers participating in the study from eight different universities and colleges. Participants in the interviews were teachers experienced in assessment of CCN students in placement. Details of the participants are listed in Table 1.

**Table 1:** Characteristics of CCN teachers

Characteristics	*N* (%)
Gender	Female: 9 (90) Male:1(10)
Part-time position (percentage position as teacher)	1 (20%) 1 (30%)
	**Mean (range)**
Age (years)	53.3 (43–65)
Experience as a teacher (years)	13.3 (0.5–28)
Experience as a CCN (years)	12.7 (1–22)

The managers of the postgraduate/master’s CCN programs in Norway were also requested to send the assessment documents for placement courses. We received assessment documents from all 10 universities and colleges by email.

### Data collection

The main data collection was from individual interviews with 10 teachers from eight different universities/ colleges, supplemented by assessment documents from all 10 universities/ colleges providing postgraduate CCN education in Norway. The interviews were conducted digitally (Zoom) from December 2021 to January 2022 due to Covid-19 pandemic restrictions. Individual interviews are considered well-suited to provide insight into the participants’ own experiences and perceptions [39, 40]. The interviews were audio recorded, had a semi-structured approach, and lasted between 44 and 69 min (average 54 min). The interview guide (Table 2) was based on themes agreed in the research group.

**Table 2:** Interview guide

1. How does the assessment of postgraduate CCN students’ clinical competence take place?
2. What do you base your assessment of the students on?
3. How do you experience the collaboration between student, teacher, and preceptor in the assessment situation?
4. What is your experience with the use of assessment documents?
5. How do you perceive correspondence between learning outcome descriptions in assessment documents and necessary competence in practice?
6. How can the assessment of postgraduate CCN students’ competence in placements be improved?

The first author conducted the interviews, made field notes, and transcribed the interviews verbatim. The third author assisted during the first two interviews that originally were pilot interviews. The quality of the pilot interviews was sufficient for them to be included in the data set. According to Malterud [41] information power is dependent on aim and design, and a larger sample size is needed for studies with broader aims. However, this study had a specific aim and the interviews provided rich data to answer the research questions, and information power was evident [41].

As a supplementary data collection, assessment documents from 10 universities/ colleges in Norway were collected between January 2021 and December 2022. The first author made a summary of the assessment documents as presented in Table 3.

**Table 3:** Assessment documents (Norwegian universities and colleges)

University/ college	Assessment form design	Document origin	Assessment form content	Learning objectives	Additional information
**1.**	Document for students’ self-evaluation. The student and preceptor can write a summary of the learning process.	Local	**Vague assessment criteria.** *Knowledge *Skills *General competence	Learning outcome descriptions for each placement period. Not specified to different units. In addition, students write personal goals for placement period.	Students write a continuous learning log where reflection on practice and their learning process is emphasized. Checklist for knowledge and practical skills
**2.**	RESPONS assessment tool The expected level of independence is marked in the assessment forms for each placement period.	RESPONS*	**Clear assessment criteria** *Patient-oriented intensive care nursing *Ethics, communication, and interaction *Professional management, quality, and patient safety	Learning outcomes are specified to learning situations and activities (with different examples).	A guide for various levels (5 levels). Assessed in relation to degree of independence.
**3.**	Document for students’ self-evaluation. The preceptor and teacher comments on the student’s learning process.	Local	**Vague assessment criteria** *Ethical and legal aspects *Communication/interaction	Learning focus related to pathophysiology, critical illness, and health status. Students write personal goals for placement period.	Placement is connected to a theoretical subject
**4.**	Document for students’ self-evaluation. Students assessed as; lower than expected, as expected. The preceptor and teacher comments on the student’s learning process.	Local	**Clear assessment criteria** *Knowledge *Skills *General competence	Different forms for different wards; ICU, intermediate care, and recovery unit.	Different forms for placement period 1, 2 and 3. Assessed in relation to degree of independence
**5.**	Separate form for each placement period. Students assessed as ‘below expected’ or ‘expected’ according to different competencies. The student, preceptor and teacher can add comments	Local	**Clear assessment criteria** *Therapeutic function *Critical care nursing *Administrative function *Teaching and professional development function	Students write personal goals for placement period in addition to the university’s learning outcomes specified for the intensive care-, intermediate- and recovery unit.	Placement courses integrated with theory course, has common learning outcomes Checklist for practical skills
**6.**	Separate form for each placement period. Students assessed “as expected” or “not as expected”. The student and preceptor write a summary of the learning process	Local	**Vague assessment criteria** *Knowledge *Skills *General competence	Students write personal goals for placement period in addition to the university’s learning outcomes	Placement 1: Basic intensive care nursing Placement 2: Co-responsibility for intensive care nursing. Placement: 3 Competent intensive care nursing
**7.**	Document for students’ self-evaluation. The preceptor and teacher can make comments on the student’s learning process	Local + NINTS**	**Vague assessment criteria** “Self-assessment” and “will continue to work on” based on a checklist for learning situations.	Students write personal goals for placement period in addition to the university’s learning outcomes.	Assessed in relation to degree of independence. Non-technical skills (NINTS**) included in assessment documents.
**8.**	Assessment form for 1- and 2-year. Students assessed at; inadequate, good, and very good level. Students write self-evaluation, preceptor and teacher add comments	Local (Inspired by AssCE***) + NINTS**	**Clear assessment criteria.** *Knowledge *Skills *General competence	Learning outcomes specified for the intensive care- intermediate- and recovery unit. Examples of inadequate, good, and very good competence level	Assessed in relation to degree of independence. Non-technical skills (NINTS**) included in assessment documents Checklist for knowledge and practical skills
**9.**	Document for students’ self-evaluation according to: «Comments/overall rating” and “continue to work with” and “conclusion”.	Local	**Vague assessment criteria**	Students write personal goals for placement period	Students write a continuous learning log where reflection on practice and their own learning process is emphasized.
**10.**	Document for students’ self-evaluation. Daily/weekly registration of learning situations.	Local + NINTS**	**Clear assessment criteria** *Situational awareness *Decision-making *Task solving *Teamwork *Creating security and trust	Students write their own goals based on the learning outcomes	Bjørk’ s model of technical skills (5 steps) and non-technical skills (NINTS**) included in assessment documents

*RESPONS: a learning and assessment tool that facilitates continuous feedback https://www.responssykepleie.no/. **NINTS: is based on Anesthetists’ Non-Technical Skills (ANTS) and is a systematic framework for assessing the non-technical skills of intensive care nurses. ***AssCE: Assessment of Clinical Education https://www.hig.se/Ext/En/University-of-Gavle/Organisation/Akademier/Faculty-of-Health-and-Occupational-Studies/Student-information/Assessment-form-AssCE.html

### Data analysis

Data from the interview transcriptions were the main data source and were analyzed using thematic analysis, which is a method for identifying, analyzing and reporting patterns in qualitative data [42]. This method is suitable to describe and show patterns in the semantic content. We used a modified version of Braun and Clarke`s [42, 43] step by step thematic analysis: (1) familiarizing, (2) coding, (3) generating initial themes, (4) reviewing and developing themes, refining, (5) defining and naming themes, and (6) writing the report. Step 1: The audio tapes were listened to, after which the interviews were transcribed by the first author. Step 2: Coding was performed focusing on the research questions to sort the data. The first author made codes by color-marking important features in the text transcripts and sorting data relevant to each code. Step 3: Initial themes subsequently emerged, as a back-and-forth approach was applied when the research team met several times to discuss the codes and identify themes. Step 4: We used an inductive approach, as we identified themes that were explicit, or recognized the surface meaning of the data. Step 5: Finally, these themes were reviewed to determine whether there were any new themes. An extract of the data analysis process is presented in Table 4.

**Table 4:** Extracts from thematic analysis (Braun & Clarke, 2006) with themes identified from interviews and assessment documents

Generating initial codes by marking interesting features of the data set.	Searching for potential themes	Reviewing themes	Defining and naming themes
“In order **to get a good assessment, the students must be assessed based on specific learning outcome descriptions, or specific goals**” [4] “I have to constantly work on **making the conversation about the student, not about the situation and the patient and relatives and others**.” [7]	Clear criteria provide assessment support and learning focus		
“It is important to have **learning outcome descriptions that they can identify with**, and where there is **not too much of a difference between school and practice**.” [3] “**The assessment criteria provided very good evaluation support**, and assurance of quality so that there is not much room for subjective assessments. It becomes a bit more like **we all speak the same language**.” [1]	To speak the same language	Clear and relevant criteria provide necessary assessment support	A need for common, clear and relevant assessment criteria
Assessment documents (Table 3): Some documents have **clear assessment criteria** that provide a guide for various levels in relation to degree of independence. Other documents have **vague assessment criteria**, mainly based on the students’ own goals for the placement period. Most documents contain learning outcomes that are based on a national framework and presented in **academic language**. **Most documents appear different in layout and content** and are mostly made locally at the universities/ colleges.	Variations in content of assessment documents		

Findings from the assessment documents (Table 3) were discussed by the research team and provided a supplement to the analysis of the main data collection. Verbatim quotations from the interviews exemplified our interpretations as reflected in the findings. Thematic analysis was considered the most appropriate method as it is descriptive and flexible [42, 43].

### Ethical considerations

The study was approved by the Norwegian Centre for Research Data on 30.09.21 (case No. 949,642). The teachers’ participation was voluntary, and written informed consent was obtained from all participants. Confidentiality, anonymity, and the right to withdraw from the study without any consequences was guaranteed. The data were recorded using an approved digital tool. Furthermore, the data material was kept secure throughout the research process in accordance with the university’s laws and regulations.

## Results

**Fig. 1:**
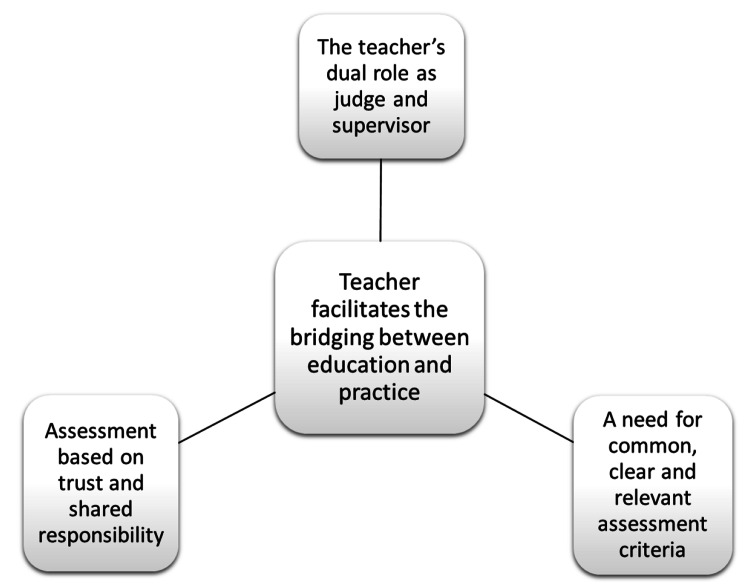
Overview of themes

Analysis of the interviews and documents led to the identification of one main theme: “Teacher facilitates the bridging between education and practice”. Placement in postgraduate nursing education is the bridge between education and practice, and we found that teachers contribute to facilitate this bridging. Further three themes were identified describing the assessment of CCN students’ competence from the teachers’ perspective and content of assessment forms: “Assessment based on trust and shared responsibility “The teacher’s dual role as judge and supervisor,” and “A need for common, clear and relevant assessment criteria”. An overview of the themes is presented in Fig. 1.

### Assessment based on trust and shared responsibility

The teachers expressed that they mostly based their assessment of the students’ competence on the written documents and dialogue with the students and preceptors in the assessment meetings. The teachers felt highly dependent on feedback from the preceptors, and it was important that the preceptors did not dispute what the students conveyed. Sometimes the teachers experienced a great discrepancy between the assessment comments from the students and the preceptors. When this occurred, the teachers would take other elements into consideration, such as their own perception of the students gained from reflection group meetings, simulation training, and other meetings. Some of the teachers found it challenging to assess the students without having seen them much in action:

“*The organization and structure around it (…) is much more problematic than the assessment form itself. Personally, I could imagine seeing the students a lot more*.” (T6).

To compensate for this drawback, the teachers talked about the importance of knowing their students well, and that they preferred to follow the students for several placement periods. The teachers expressed that it is necessary to have clear criteria and the students must always be assessed based on the learning outcomes. The teachers also described how dependent they were on having preceptors who documented what wasn’t a satisfactory level of competence. If the students were at risk of failing the placement course, the teachers explained that their role was to clarify what they expected the student to achieve. Furthermore, our findings suggest that some of the teachers felt responsible for the final assessment of the students:

“*It happens that we do not agree with the student’s level, and so on. But then it is quite clear in our documents (…) that it is the teacher who has the last word.*” (T5).

The teachers assumed that it could be more difficult for the preceptors to set strict requirements for the students because a teacher has a different and somewhat more distant perspective. However, the teachers experienced that most of the preceptors took their role seriously and did not refuse to fail students. They found that most of the preceptors took great pride in being CCNs and expected the CCN students to maintain a certain standard. The learning outcomes were very much governing, but the preceptors also had an inner pride to help their students perform as well as possible. They looked upon themselves as kind of gatekeepers, because the students will probably become their future colleagues and must be ready to function as competent CCNs when they start working.

There is great pressure to educate more CCNs, and the teachers were concerned about the preceptor’s lack of time to supervise and assess students properly. Sometimes the focus was more on quantity than quality in the guidance. Despite the teachers concern about the preceptors’ lack of time, some of the teachers expressed their deep respect for the preceptors as they considered them to have high moral and ethics, and they looked well after the students in placement.

### The teacher’s dual role as judge and supervisor

We found that the teachers experienced a kind of duality in the role as teachers. They had to both look after the student and support their learning process, and at the same time ensure high quality of the future CCNs. The teachers also experienced having to guide and support the preceptors in their role. This was especially evident in the assessment of underachieving students because the preceptors felt it very unpleasant to be involved in failing a student:

“*We stand there and must be, in a way, the student’s lawyer and the preceptor’s lawyer or everyone’s support, so it is demanding*.” (T3).

The teachers would then provide guidance on the assessment based on the learning outcome descriptions in the assessment documents. They would also make sure to adequately document the assessment of the student. Having a close collaboration with the preceptors and the nursing managers in the ICU made the duality of the role less demanding. There is a need for the teachers, students, and preceptors to collaborate closely, and we found that the pedagogical role of the teacher could influence the students’ learning process in placement:

“*I think it’s important for learning, that they are confident in me as a teacher and see that I want them well. That doesn’t mean all students should pass. But at least it gives a good relationship with the students, and it gives them a broad and fair assessment, and that’s important to me.” (T5)*.

Furthermore, the teacher seemed to provide other elements to the assessment process than the preceptor because teachers have a different perspective.

The teachers in our study emphasized that it was essential to clarify expectations and to establish a close relationship where the threshold for communication with each other was low. One of the success factors for a good collaboration was that they agreed on what should be the student’s focus. On the other hand, the lack of continuity in follow-up in placement affected the learning and assessment process negatively. The teachers also described how some preceptors could make the students feel insecure if the students felt that they were constantly in an assessment situation. Further, several of the teachers found it important to be updated in practice:

“*The ideal would have been if, as a CCN teacher, you could have worked 50/50. This way you could both have been a part of the teaching staff and a part of the patient-oriented staff. Then you could have kept up to date*.” (T8).

Two of the participants worked part-time as teachers and CCNs in practice. They appreciated that this dual role might be positive for the collaboration with the university and the hospital. In addition, the teachers felt more available to the students and preceptors, which seemed to be an advantage in the process of assessing the students’ competence.

### A need for common, clear and relevant assessment criteria

We found that there is great variation in the assessment criteria for CCN placement education in Norway as shown in Table 3. Some of the assessment documents were clear and relevant to practice, whereas others were indistinct. Most of the universities and colleges had developed their own assessment documents, and the content of the documents varied much depending on the different educational institutions (Table 3). The assessment form content was defined as “clear” if the learning outcomes were specified to different learning situations and activities and characterized as “vague” if the learning outcomes were scarcely described.

Accordingly, some of the teachers experienced that the assessment forms were reliable and helpful in the assessment situations:

“*The assessment criteria provided very good evaluation support, and assurance of quality so that there is not much room for subjective assessments. It becomes a bit more like we all speak the same language*.” (T1).

If the goals were clear, it was easier to give example of how far the students had come in achieving their goals. However, other teachers experienced that the assessment form formulations were too unclear to reflect the defined standards. If both the students and preceptors failed to clearly set out what the expected level was, the teachers had to translate the learning outcomes to make them more manageable. The need for preceptors to be clear was especially important if the students were at risk of failing. The teachers emphasized that the assessment criteria were very important to clarify which competencies the students needed to improve:

“*I have to constantly work on making the conversation about the student, not about the situation and the patient and relatives and others*.” (T7).

The use of clear competence descriptions with a focus on non-technical skills could be helpful in separating the student’s personality from the student’s skills. The teachers experienced that assessment documents could be decisive for what became the learning focus during placement. There is a need for a common understanding, and it was important that the preceptors read and familiarized themselves with the educational plan and the learning outcome descriptions.

How actively the preceptors and students used the assessment documents varied. The teachers were frustrated regarding the preceptors’ commitment and how much time they had to familiarize themselves with it. Sometimes the preceptors had a different opinion on what the student should learn, and this could then affect the assessment of the student:

“*It is important to have learning outcome descriptions that they can identify with, and where there is not too much of a difference between school and practice*.” (T3).

Some of the teachers tried to make the assessment criteria from the university consistent with the expected level of functional ability and had operationalized the learning outcomes for placement courses in collaboration with CCNs at the hospital. Even though clear assessment criteria were highly appreciated by the teachers, some were worried about making things too specific. They found it important that the students should clarify their own goals to better see and understand what they needed to learn.

Our findings from the interviews and documents indicate that which competencies are emphasized vary. According to the teachers, the students found managing technical skills most important. Holistic nursing and non-technical skills such as communication and anticipate and stay ahead of the situation, was highly emphasized by the preceptors. These competencies were also regarded as important by the teachers. In addition, they regarded ethics and attitudes, knowledge, linking theory to practice, and working according to evidence-based practice as important competencies. The teachers also highlighted the need to strive for a balance between practicality and academic knowledge, and the importance of students’ self-reflection and self-assessment.

It is necessary to document the students’ competence level, but we found that there is a considerable variety regarding how and what is documented in relation to placement courses. Some of the teachers commented on the possibility of using a digital assessment tool. They presumed that the use of digital assessment tools would be a great improvement, because it would probably make the assessment documents more available and transparent for both students, preceptors and teachers. Further we found that the teachers were generally interested in new innovative methods to improve the assessment of CCN students’ competence in placement.

## Discussion

This study aimed to describe how teachers experienced the assessment of CCN students’ competence in placement. Additionally, to explore the content of assessment documents used for CCN placement education in Norway. Findings from the interviews indicate that teachers found it important but challenging to assess CCN students’ competence. The complexity of assessment of nursing students’ competence in placement is a matter of concern in nursing education and has been stated in several previous studies [10, 12, 13, 32]. Additionally, it can be even more challenging to assess what nursing competence is at postgraduate level [18, 19, 24]. In Norway, the CCN students are experienced registered nurses, and some students have work experience from ICU prior to starting the CCN education. According to Solberg et al. [5] a master’s programme for nurses in critical care is intended to cultivate nurses who are able to integrate advanced theoretical knowledge with practical and interpersonal skills in caring for critically ill patients. Teamwork, decision-making, to manage situations and care for patients beyond the technical aspects, showing personal maturity and have a good attitude are terms used to describe professional competence in critical care nursing [5, 7, 11]. These competencies should be described in assessment documents to help distinguish the competence of a qualified nurse from a qualified CCN. According to Mårtensson et al. [19] a structured assessment tool that includes behavior cues could help teachers and preceptors improve the clarity of their assessment and feedback to students at postgraduate level.

This study is the first to map the content of the assessment documents for CCN placement education in Norway. Even though there is a national framework for CCN education, we found that each educational institution had different assessment documents and there is great variation in the assessment criteria for placements as shown in Table 3. Some of the assessment forms were indistinct, whereas others were clear and relevant to practice. Assessment instruments developed according to evidence-based practice and validated are not being used, as each educational institution make its own instruments. The inconsistency in assessment methods and instruments both between higher education institutions and between countries has been stated in previous research [2, 13, 30], and our study shows that even in a small country like Norway there is not a common national assessment form for CCN education.

Some of the teachers found that the assessment instruments provided good assessment support. In particular, the assessment criteria with a focus on the non-technical skills could be helpful in separating the students’ personality from the students’ skills. However, other teachers experienced that they had to translate the criteria to make them manageable. This made the teachers concerned about getting the preceptors to talk about the students’ competence rather than the situation. The purpose of assessment is to provide feedback to the students on their ability based on their learning outcomes [10], and the use of clear competence descriptions is both necessary and important to give the students a fair assessment [24].

The teachers in our study experienced that learning outcomes in assessment documents could be decisive for what became the learning focus during placement. However, focus varied depending on how actively the preceptors and students used the assessment documents. The teachers were frustrated about some of the preceptors’ lack of commitment, and sometimes the preceptors even had a different opinion on what the student should learn. This is in line with previous research on the formal assessment discussions in placement [14]. An interesting finding in our study was that some of the teachers had operationalized the learning outcomes for placement in collaboration with CCNs working at the hospital. Constructive alignment is important if there is a need for students to integrate and apply theory into practice [22, 23]. The assessment criteria must be consistent with the expected level of functional ability and should be developed in collaboration with practice.

We found that the teachers based the assessment on different methods such as students’ self-assessment, the preceptors’ feedback, and the teachers’ perception of students from meetings and written assignments. Although they trusted the students’ and preceptors’ feedback, they preferred to get to know the students well to be able to form their own opinion about the students’ competence level. This finding is consistent with Helminen et al. [32] who highlighted that support from the teacher during the assessment process was relevant both for the students and the preceptors. Findings from our study indicate that the teachers’ presence in placement is important. This is an interesting finding since the role of nursing teachers has changed from a clinically skilled practitioner to a more distant role in the placement context [33–35]. Nevertheless, other studies support this finding and underline the value of teachers pedagogical and academic contributions by providing a different perspective in the assessment process [33, 36].

Self-reflection, balancing practicality and theoretical knowledge, and working according to evidence-based practice were competencies highlighted by the teachers. Benner [44] states that these are among the core competencies in advanced critical care nursing. Even so, the teachers experienced that they emphasized different competencies than the students and preceptors in the assessment process. Nurse educators are positioned to facilitate opportunities for students and practicing nurses to be involved in evidence-based practice care initiatives. These competencies are important to make the students capable of further development in the field of critical care nursing [45]. Cant et al. [46] found that some students were more satisfied with the role of the teacher than preceptors because of their ability to integrate theory and practice and stimulate students’ critical thinking. A holistic approach to the term competence involves the student’s ability to use theory, judgment, critical thinking, and professionalism [7, 19, 47]. Thus, it’s important to assess both the student’s technical and non-technical skills in placement.

Most of the teachers in our study were employed by educational institutions, and some suggested that it would have been ideal if they could work 50/50 as teachers and CCNs in the patient-oriented staff to be updated in practice. Two of the participants in this study worked in joint positions at the university and the hospital. They could observe and meet the students more frequently, which was valuable in the assessment process. Also, the teachers appreciated how this could be positive for the collaboration between the university and hospital in general. This finding aligns with those of previous studies suggesting that educators in joint positions could strengthen the clinical learning environment for students [8, 16]. Mathisen et al. [8] states that nurse educators who are insiders in both settings, are ideally placed to contribute to bridge the theory-practice gap. Further, partnerships between academia and practice can lead to improved patient care and health system innovations [45]. However, we found that there were some challenges related to having two employers. For one thing, they must be clear about when they are teachers and dedicated to taking care of the students, and when they are CNNs focused on taking care of patients.

Findings in our study suggest that the teachers looked upon themselves as responsible for the assessment process. However, the teachers also regarded the assessment as a shared responsibility. The teachers felt they had a common understanding with the preceptors regarding the importance of providing high-quality care to critically ill patients. Furthermore, the teachers experienced that the preceptors looked upon themselves as gatekeepers, and that they are in a position to shape and approve who is most likely to become their future colleagues. This finding is supported by other studies [10, 12, 13].

Further, we found that the teachers could experience a duality in their role. They had to support the students’ learning process and at the same time ensure an adequate competence level. This could sometimes be difficult, especially if the students were at risk of failing. The teachers cared for the students, and in nursing education, ethos and core values are important to become a professional and caring nurse. The term “failing to fail” is described as nursing faculty members struggling to assign failing grades to underperforming students in the clinical setting [48], and involves a difficult conversation that requires confidence and could cause emotional harm to both the student and educators. Thorup et al. [49] states that a nurse must have sensitivity in order to be able to relate to other people, and vulnerability is thus an important resource in nursing. Nevertheless, the teacher must ensure safe and high-quality care for the patients. The sense of care they have in relation to their students must not affect their professional responsibility as teachers. However, belief in students’ ability to grow is seen as central foundation and condition. This perspective is valuable, especially in the formative assessment of the students [21], and the teacher’s pedagogical competence is essential in this matter.

Findings in this study suggest that the teachers could provide guidance because they had a more distant perspective of the students and were more familiar with the assessment criteria. Moreover, the teachers could facilitate the bridging between the education and working life by linking theory and practice. Pedagogical competence is also important to guide the preceptors in their role, especially in the assessment of underachieving students. This is in line with previous research stating that the educational institutions contribute to the bridging between theory and practice [6–8].

The teachers in our study were concerned about the preceptor’s lack of time to supervise and assess students, which has also been stated in other studies [14, 36]. The pressure to educate a growing number of students in placement can lead to additional strain on the CCNs [24, 50]. According to Järvinen et al. [36] the lack of time and the increase in number of students affected the ability to assess the students properly during placement. Nurses’ competence is crucial to achieve the goal of providing safe and high-quality care [3, 4]. Patient care must always be prioritized, but the increasing pressure to educate more CCNs may lead to placement periods of shorter duration and less follow-up, which could affect the qualifications of the future CCNs. We believe that teachers have a key role in providing educational quality in placement due to their responsibility for the assessment process of students.

### Methodology considerations

Regarding data collection and analysis, the provision of trustworthiness, credibility, dependability, confirmability, and transferability must be ensured [23, 28]. A pilot interview was thus performed to ensure credibility in data collection. The first steps of the analysis were mainly done by the first author, but to ensure credibility, the identified themes were discussed and approved by the research team. Credibility was also strengthened by ensuring that actual statements from the participants were represented in the manuscript. Transparency was maintained throughout the process, with a record being kept of all stages of data collection and analysis. Some of the text from the transcripts was read by the whole research team to assess comparability to the codes and themes derived by the first author. Transferability was attended to by conducting interviews with CCN teachers from eight different universities and colleges in Norway, and dependability was ensured by using the same interview questions for all participants. Dependability was also strengthened by the researcher’s experience as a CCN and preceptor, which provided a deep understanding of assessment of students’ competence. The authors who performed the interviews were teachers at the same university as two of the participants. This could be a limitation as this might have influenced the participants to speak less freely. But it can also be an advantage to have knowledge about the culture being studied, whereas it can be a challenge to create a distance when analyzing the material [23, 28]. Confirmability was ensured by using representative quotations to illustrate information in relation to the findings. By providing descriptions of the participants and data collection, the transferability of our findings to another context was enhanced. A limitation of this study could be that the participants evaluated their own role. Further, the use of Zoom could be a limitation, as this may have affected the quality of the interviews. However, the researcher who conducted the interviews experienced few disturbances due to the use of Zoom. This study was carried out on a relatively small sample of teachers in Norway and may not reflect the teacher role in other countries. A strength of this study is that we included participants employed by different universities that were geographically spread out within the country. Additionally, we gathered assessment documents from all universities and colleges in Norway providing CCN education.

## Conclusions

We found that teachers have a key role in the assessment of students in placement, and it is important that they are present in the clinical setting. Teachers in our study experienced that the assessment of CNN students’ competence in placement can be challenging and complex. Nevertheless, they found their role in the assessment of students’ competence to be important to ensure high-quality care for patients. The teachers contributed valuable support and guidance to both the students and preceptors in the assessment meetings during placement. Collaboration in the education of CCNs is essential, and nursing teachers can facilitate the bridging between education and working life by linking theory to practice, promoting critical thinking, and working according to evidence-based practice. Assessment documents and criteria vary, and this study underpins the notion that common, clear and relevant assessment criteria are essential for learning focus and provide valuable assessment support. Teachers and preceptors must continue to work together, to improve and develop reliable and effective assessment strategies. CCN students need constructive feedback on their skills and performance to maintain high-quality nursing education in placement at advanced level.

### Implications for practice and research

We believe that teachers employed in joint positions between education and practice could further enhance the collaboration between practice and education institutions regarding education of postgraduate CCNs. We further suggest the development of common national assessment documents for CCN education. The assessment documents should be developed as a collaboration between education and practice to meet the constant changes in critical nursing care and treatment, and the assessment documents should be constantly evaluated and improved. Research and innovation regarding development of user friendly and available assessment tools, like digital assessment tools, is also needed. Further research on assessment of CCN students’ competence from the students’ and preceptors’ perspective is recommended.

### Electronic supplementary material

Below is the link to the electronic supplementary material.

Supplementary Material 1

## Data Availability

The data from the audio-recorded interviews used to support the findings of this study are available from the corresponding author upon reasonable request.

## Abbreviations

**CCN**: Critical care nursing
**CCNs**: Critical care nurses
